# UMONS-TAICHI: A multimodal motion capture dataset of expertise in Taijiquan gestures

**DOI:** 10.1016/j.dib.2018.05.088

**Published:** 2018-05-23

**Authors:** Mickaël Tits, Sohaïb Laraba, Eric Caulier, Joëlle Tilmanne, Thierry Dutoit

**Affiliations:** a Numediart Institute, University of Mons, Belgium; b University of Nice Sophia Antipolis, Nice, France

## Abstract

In this article, we present a large 3D motion capture dataset of Taijiquan martial art gestures (*n* = 2200 samples) that includes 13 classes (relative to Taijiquan techniques) executed by 12 participants of various skill levels. Participants levels were ranked by three experts on a scale of [0–10]. The dataset was captured using two motion capture systems simultaneously: 1) Qualisys, a sophisticated optical motion capture system of 11 cameras that tracks 68 retroreflective markers at 179 Hz, and 2) Microsoft Kinect V2, a low-cost markerless time-of-flight depth sensor that tracks 25 locations of a person׳s skeleton at 30 Hz. Data from both systems were synchronized manually. Qualisys data were manually corrected, and then processed to complete any missing data. Data were also manually annotated for segmentation. Both segmented and unsegmented data are provided in this dataset. This article details the recording protocol as well as the processing and annotation procedures. The data were initially recorded for gesture recognition and skill evaluation, but they are also suited for research on synthesis, segmentation, multi-sensor data comparison and fusion, sports science or more general research on human science or motion capture. A preliminary analysis has been conducted by Tits et al. (2017) [1] on a part of the dataset to extract morphology-independent motion features for skill evaluation. Results of this analysis are presented in their communication: “Morphology Independent Feature Engineering in Motion Capture Database for Gesture Evaluation” (10.1145/3077981.3078037) [1]. Data are available for research purpose (license CC BY-NC-SA 4.0), at https://github.com/numediart/UMONS-TAICHI.

## Specifications Table

**Table:** 

Subject area	*Human movement science*
More specific subject area	*Sports science, gesture recognition, synthesis, segmentation and evaluation, sensor comparison*
Type of data	*3D Motion Capture data, sampled at 179 Hz (Qualisys), and 30 Hz (Kinect)*
How data were acquired	*Qualisys optical motion capture system (11 Oqus cameras), Microsoft Kinect v2*
Data format	*Corrected, completed, filtered, annotated, segmented (.c3d,.tsv,.txt)*
Experimental factors	*Skill, Taijiquan techniques, morphology*
Experimental features	*Twelve participants with different levels (ranked on a scale of 0–10) performed a total of 2200 Taijiquan gestures (divided in 13 different gesture classes).*
Data source location	*Mons, Belgium*
Data accessibility	https://github.com/numediart/UMONS-TAICHI

## Value of the data

• Large and various dataset (2200 samples, 12 participants, 13 classes).
• Both high quality data (68 markers with < 1 mm spatial accuracy, 179 Hz), and low-cost data (Microsoft Kinect v2 skeletal data).
• Data manually corrected and annotated, automatically gap-filled and filtered.
• Participants skill levels were ranked by three teachers (on a scale of 0–10).
• Relevance to the fields of human movement science, gesture recognition, synthesis and evaluation, movement segmentation, and multi-sensor data comparison and fusion.

## 1 Data

This brief article presents a multimodal motion capture (MoCap) dataset of Taijiquan martial art gestures. The data were initially recorded for gesture recognition and skill evaluation. The dataset includes 2200 sequences of 13 classes (relative to different Taijiquan techniques) performed by 12 participants of different levels of expertise. Participant levels have been ranked by three Taijiquan teachers (on a scale of 0–10). The dataset contains both unsegmented and manually segmented sequences. The data were captured using the Qualisys optical motion capture system and the second version of the Microsoft Kinect simultaneously. The Qualisys motion capture system used consists of 11 high-speed infrared cameras that track 68 retroreflective markers placed over the performer׳s body, at a frame rate of 179 Hz. The Kinect sensor, on the other hand, is a low-cost time-of-flight depth sensor that estimates 25 3D joints locations at a frame rate of approximately 30 Hz. A subset of this dataset has already been used in a previous research [1] to validate a method of morphology independent feature extraction in MoCap data for skill evaluation.

To the authors׳ knowledge, it is the first dataset of sports gestures comprising simultaneously a large number of participants (12), a large number of different classes (13), and a variety of skill levels, and captured with two different motion capture systems.

## 2 Experimental design, materials and methods

### 2.1 Participants

Twelve participants volunteered to participate in the dataset recordings. All of them attended courses in the Taijiquan school Eric Caulier,^1^ and were assigned a category according to their level: Novice, Intermediate, Advanced or Expert (three teachers of the school). Each Taijiquan teacher also provided individual rankings for each participant, on a scale of 0–10. These rankings were provided independently by each teacher, from their personal knowledge of all the participants during courses.

Relevant personal details for each participant, including age, height, weight, gender, practice experience and skill level can be found in Table 1.

**Table 1:** Personal details of participants. Skill was ranked with a score between 0 and 10 by three teachers. Each one of their rankings, as well as their mean (Skill_µ_) is indicated in this table. All participants attended courses in the Taijiquan school Eric Caulier, and were assigned a category according to their level (Novice, Intermediate, Advanced or Expert).

ID	Gender (M/F)	Age	Weight (kg)	Height (cm)	Practice (year)	Category	Skill_1_ (0–10)	Skill_2_ (0–10)	Skill_3_ (0–10)	Skill_µ_ (0–10)
P01	M	56	95	196	32	Expert	9.3	9	10	9.43
P02	F	57	78	163	30	Expert	9.6	9.1	10	9.57
P03	F	62	58	162	24	Expert	8.5	8.5	9	8.67
P04	F	47	53	150	12	Advanced	8.2	8	8	8.07
P05	F	71	61	163	14	Advanced	6.8	7.4	7.5	7.23
P06	M	25	76	180	10	Advanced	8.4	8.6	8.5	8.5
P07	F	49	57	157	4	Intermediate	7	6.8	6.5	6.77
P08	F	34	56	158	3	Intermediate	8	7.3	7	7.43
P09	M	51	90	178	2.5	Intermediate	6.9	6.8	6.85	6.85
P10	F	59	55	163	1	Novice	6	5.8	6.5	6.1
P11	F	65	58	165	0.2	Novice	5	4.9	5	4.97
P12	M	28	96	181	0.6	Novice	5.8	6	5.75	5.85
M		50.33	69.42	168	11.11		7.46	7.35	7.55	7.45
SD		14	15.93	12.46	11.15		1.37	1.29	1.53	1.38

### 2.2 Recording protocol

The Qualisys system tracked 68 retroreflective markers placed on the whole body (for detailed placement, see Table 2), with a frame rate of 179 Hz and a spatial accuracy of 1 mm. The dextrogyre coordinate system was placed on the ground, in the middle or the recording area, with the vertical axis as the *z*-axis. At the beginning of each recording, a participant was standing approximately above the origin of the coordinate system facing the *x*-axis direction. After each gesture, the participant was again approximately facing the *x*-axis direction.

**Table 2:** Marker placement. Labels and positions of 68 markers attached (scratched) to an elastic neoprene suit, according to Qualisys and C-Motion specification for standard full-body motion capture. Cluster markers (upper arm, forearm, thigh and shank) are placed approximately on the body and are only used for tracking in Visual3D™ software (C-Motion, Inc., Rockville, MD, USA).

**Marker label**	**Marker placement**
**Head markers (left and right)**	
L/RFHD	Approx. over left/right temple.
L/RBHD	Back of the head, approx. in a horizontal plane with front head markers.
**Torso markers**	
CLAV	Clavicles, located approx. at the jugular notch.
STRN	Sternum xiphoidal process.
CV7	7th cervical vertebrae.
TV10	10th thoracic vertebrae.
**Arm and hand markers (left and right)**	
L/RAC	Acromion.
L/RUA1-2	Cluster of two markers placed on the lateral surface of the upper arm.
L/R_HLE	Humerus lateral epicondyle.
L/R_HME	Humerus medial epicondyle.
L/RF1-2	Cluster of two markers placed on the lateral surface of the forearm.
L/R_RSP	Radius styloid process.
L/R_USP	Ulna styloid process.
L/R_HM1	2nd metacarpal (index).
L/R_HL5	Lateral head of 5th metacarpal (pinkie).
**Pelvis markers (left and right)**	
L/R_IAS	Anterior superior iliac spine.
L/R_IPS	Posterior superior iliac spine.
**Leg and foot markers (left and right)**	
L/R_FTC	Most lateral prominence of the greater trochanter.
L/R_TH1-4	Cluster of four markers placed on the lateral surface of the thigh.
L/R_FLE	Femur lateral epicondyle.
L/R_FME	Femur medial epicondyle.
L/R_SK1-4	Cluster of four markers placed on the lateral surface of the shank.
L/R_FAL	Lateral prominence of the lateral malleolus.
L/R_TAM	Medial prominence of the medial malleolus.
L/R_FCC	Aspect of the Achilles tendon insertion on the calcaneus.
L/R_FM1	Dorsal margin of the 1st metatarsal head.
L/R_FM2	Dorsal aspect of the 2nd metatarsal head.
L/R_FM5	Dorsal margin of the 5th metatarsal head.

**Fig. 1:**
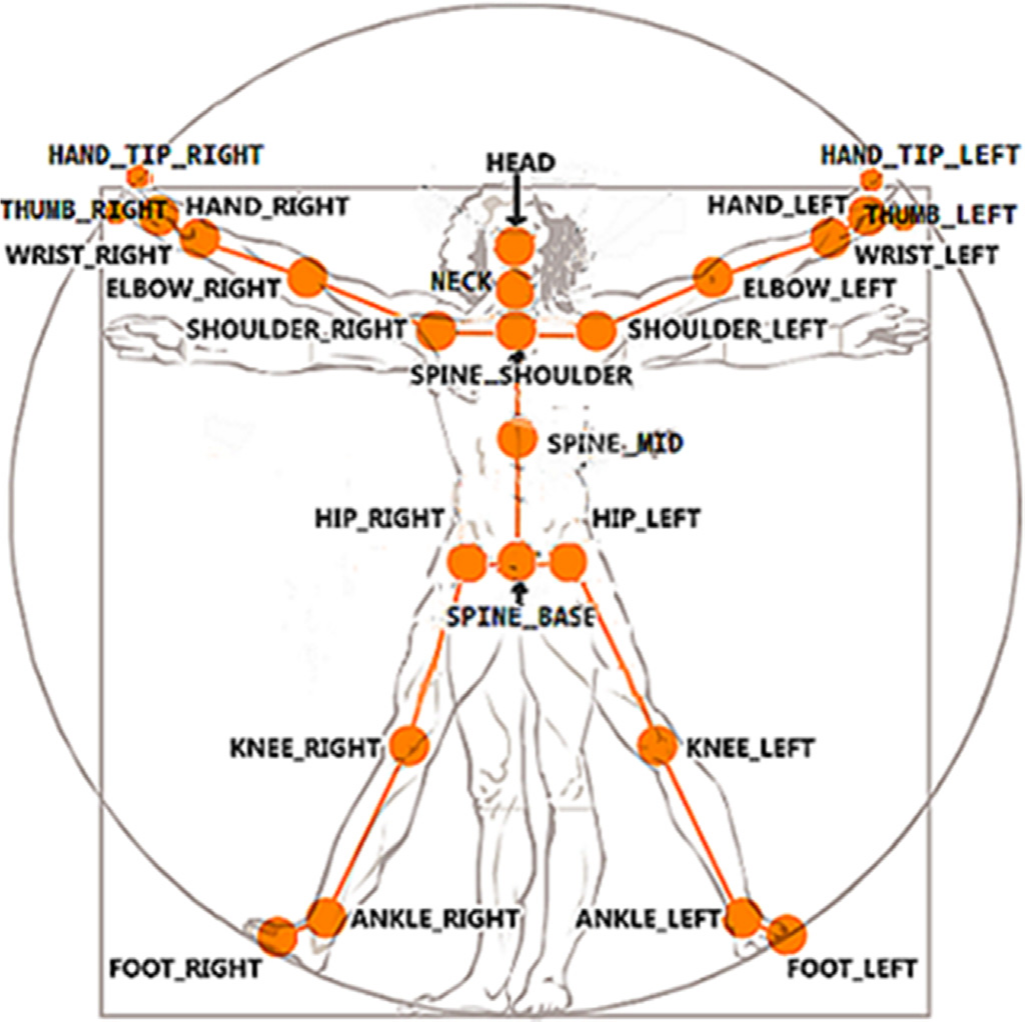
Skeleton joints positions relative to the human body.

The Kinect sensor tracked the estimated 3D locations of the standard 25 joints (Fig. 1) at a frame rate of approximately 30 Hz. As the recording frame rate of this system is not constant, the timestamp of each frame was also recorded, for synchronization purpose.

All participants performed 13 different techniques of the popular Taijiquan style ‘*Yang’*, all learned at the Taijiquan school Eric Caulier.

These techniques are divided into two main categories: the Five Exercises (*Wu gong*), composed of five simple gestures, and the Eight Techniques (*Bafa*), composed of eight more complex gestures (see details in Table 3). All techniques are described in detail in [2]. Videos of the gestures performed by a teacher are included with the dataset as supplementary information. During the recording session, each participant was asked to perform three different rendition types, as described in Table 4.

**Table 3:** Five exercises and eight techniques of the Yang Taijiquan style.

**Gesture ID**	**Name**	**Movement type**
**Five exercises (*****Wu gong*****)**		
G01	*Beginning position (Wuji)*	Static posture, symmetric
G02	*Tree posture (Taiji)*	Static posture, symmetric
G03	*Open and close lotus flower*	Symmetric
G04	*Bring sky and earth together*	Symmetric
G05	*Canalize energy*	Asymmetric (left or right)
**Eight techniques (*****Bafa*****)**		
G06	*Drive the monkey away*	Asymmetric (left or right)
G07	*Move hands like clouds*	Asymmetric (left or right)
G08	*Part the wild horse’s mane*	Asymmetric (left or right)
G09	*Golden rooster stands on one leg*	Asymmetric (left or right)
G10	*Fair lady works shuttles*	Asymmetric (left or right)
G11	*Kick with heel*	Asymmetric (left or right)
G12	*Brush knee and twist step*	Asymmetric (left or right)
G13	*Grasp the bird’s tail*	Asymmetric (left or right)

**Table 4:** Types of renditions performed by the participants.

**Type ID**	**Description of the rendition**
T01	**Five exercises** Each exercise is repeated four times in a row. After the four repetitions, a pause of 2–5 s is respected, before the transition to the next exercise. For the fifth exercise (Canalize energy), which is the only asymmetrical gesture of the sequence, the four repetitions consist of a succession of left and right side gestures, in the order: ‘left–right–left–right’.
T02	**Eight techniques** Each technique is repeated four times in a row. After the four repetitions (‘left–right-left–right’), a pause of 2–5 s if respected, before the transition to the next technique.
T03	**Chained eight techniques** Idem as the previous type, but no pause is respected during the transition between two different techniques.

### 2.3 Data processing

Qualisys MoCap data were manually corrected using the Qualisys Track Manager (QTM) software.^2^ The corrected data were then extracted in standard 3D motion data formats (C3D and TSV). All missing data (generally due to marker occlusions) were estimated with an automatic MoCap data recovery method.^3^

The Kinect data were saved into “.txt” files which contain several lines corresponding to each captured frame. Each line contains one integer number (ms), relative to the moment when the frame was captured, followed by 3 × 25 float numbers corresponding to the 3-dimensional locations of the 25 body joints.

### 2.4 Manual annotation (segmentation)

All renditions were manually labeled from Qualisys data to identify beginning and ending of each instance of a gesture. To that end, the MotionMachine framework [3] was used.

**Fig. 2:**
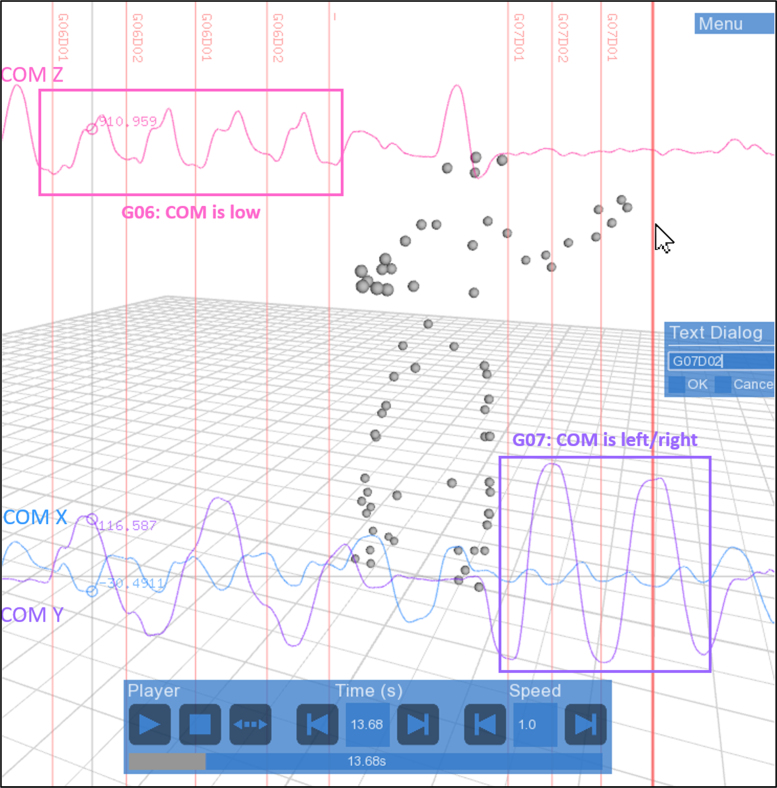
Screenshot of the annotation software. Layered display of: 1. 3D motion (gray spheres); 2. 2D-graphs showing evolution in time of the COM coordinates (blue = *x*, purple = *y*, pink = *z*); 3. Annotations (red vertical lines and labels). 4. GUI (blue windows, allowing navigation in the file, and label edition). In this example, G06 has been annotated, and G07 is being annotated. For G06, labels are placed when the *z*-axis of the COM is low, and for G07, labels are placed when the COM *y*-axis if low (COM is on the left) or high (COM is on the right).

The annotation software created from this framework^4^ allows mouse-controlled simultaneous visualization of 3D movements (Qualisys data), and 2D curves displaying temporal evolution of each coordinate of their Center Of Mass (COM), estimated from the mean position of the 68 markers. COM coordinates can be used as a global visual indication for systematic segmentation, as described in Table 5. In the software, the time of the MoCap sequence is controlled by the horizontal position of the mouse, and any mouse click creates a label at its current position. The GUI then allows the edition of the label list. Fig. 2 shows an example of the annotation procedure. In this example, gestures G06 and G07 are being annotated.

**Table 5:** Manual segmentation rules for the 13 gestures based on visual indications on direct 3D motion and COM coordinates.

**Manual segmentation rules**		
**Gesture**	**Start**	**End**
G01	(static posture)	(Static posture)
G02	(Static posture)	(Static posture)
G03	COM low.^a^	COM low.
G04	COM high.^b^	COM high.
G05	COM high.	COM low, foot take-off.
G06	COM low.	COM low.
G07	COM on one side.^c^	COM on the other side.
G08	COM back at the center^d^ (Foot take-off).	COM back at the center
G09	Foot take-off.	Foot starts to go down.
G10	COM back at the center.	COM back at the center.
G11	COM low (Just before foot take-off).	COM low.
G12	COM back at the center.	COM back at the center.
G13	Just before foot take-off.	COM back at the center.

a COM low: local minimum of COM *z*-axis.

b COM high: local maximum of COM *z*-axis.

c COM on one side: local extremum of COM *y*-axis.

d COM back at the center: local extremum of COM *y*-axis, generally near *y*-axis mean position.

From annotations, Qualisys data were automatically segmented using the MoCap Toolbox for Matlab [4] and MoCap Toolbox extension.^5^ All unsegmented files were named using the convention ‘PppTttCcc’ (e.g. P01T01C01) for which ‘pp’ is the performer ID (see Table 1), ‘tt’ is the type of the sequence (see Table 4) and ‘cc’ is the number of the clip (repetition of the same sequence). All segmented files were named using the convention ‘PppTttCccGggDddSss’ (e.g. P01T01C01G01D01S01). ‘gg’ indicates the gesture (see Table 3), ‘dd’ indicates the direction (01 for left and 02 for right – symmetric gestures are denoted D01), and finally ‘ss’ indicates the instance of the gesture (as each gesture is repeated several times during a clip).

### 2.5 Data synchronization

**Fig. 3:**
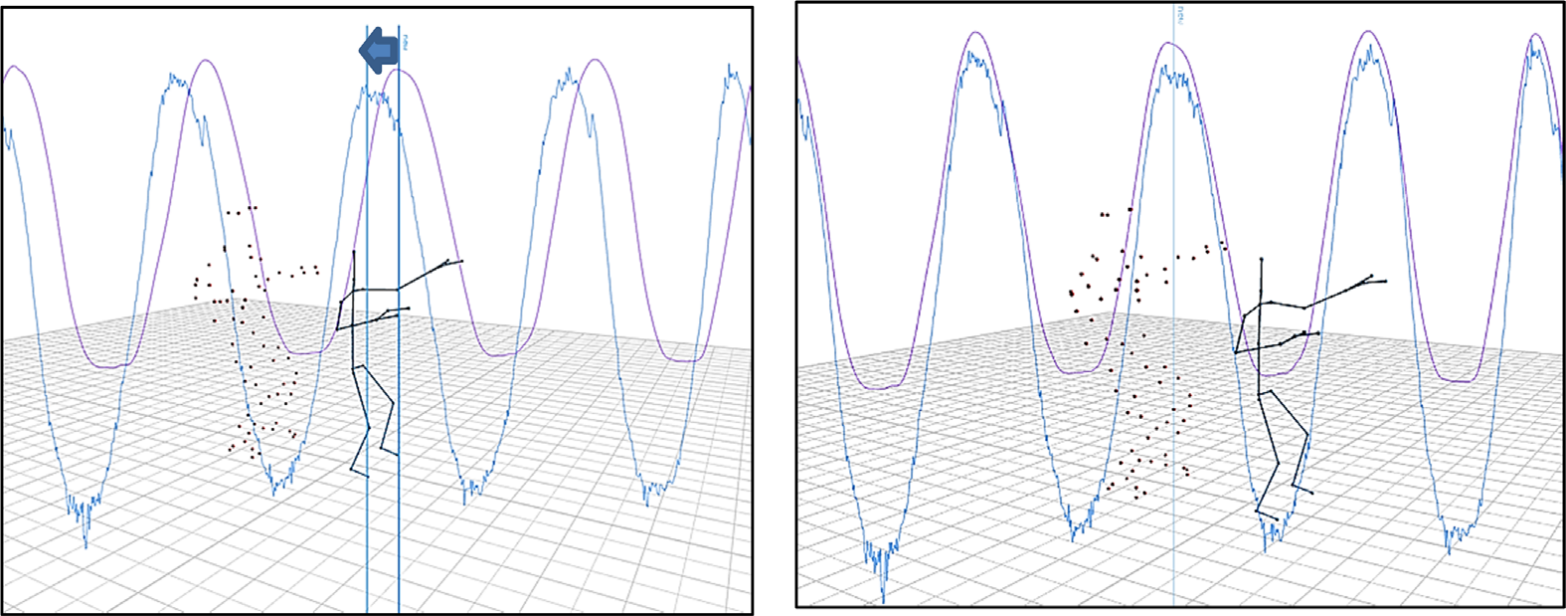
Visualization of the process of synchronization in MotionMachine framework.

The data from both Qualisys and the Kinect were synchronized with the use of the MotionMachine framework. One important feature of this framework is the management of timed sequences. This allows the synchronization of the data by means of time and not by frame indexes. For each unsegmented sequence, the delay between files was estimated using the MotionMachine framework (see Fig. 3), and the data were manually synchronized by removing the first extra frames from the longest sequence.

## Footnotes

1 Taijiquan school Eric Caulier: http://www.taijiquan.be/.

2 Qualisys Track Manager: http://www.qualisys.com/software/qualisys-track-manager/.

3 MocapRecovery: https://github.com/numediart/MocapRecovery.

4 Code accessible at: https://github.com/numediart/ofxMotionMachine/tree/master/mmTutorial_4_Annotation.

5 MoCap Toolbox Extension: https://github.com/numediart/MocapRecovery/tree/master/MoCapToolboxExtension.
